# Combining classifiers for improved classification of proteins from sequence or structure

**DOI:** 10.1186/1471-2105-9-389

**Published:** 2008-09-22

**Authors:** Iain Melvin, Jason Weston, Christina S Leslie, William S Noble

**Affiliations:** 1 NEC Laboratories of America, Princeton, NJ, USA; 2 Computational Biology Program, Sloan-Kettering Institute, Memorial Sloan-Kettering Cancer Center, New York, NY, USA; 3 Department of Genome Sciences, Department of Computer Science and Engineering, University of Washington, Seattle, WA, USA

## Abstract

**Background:**

Predicting a protein's structural or functional class from its amino acid sequence or structure is a fundamental problem in computational biology. Recently, there has been considerable interest in using discriminative learning algorithms, in particular support vector machines (SVMs), for classification of proteins. However, because sufficiently many positive examples are required to train such classifiers, all SVM-based methods are hampered by limited coverage.

**Results:**

In this study, we develop a hybrid machine learning approach for classifying proteins, and we apply the method to the problem of assigning proteins to structural categories based on their sequences or their 3D structures. The method combines a full-coverage but lower accuracy nearest neighbor method with higher accuracy but reduced coverage multiclass SVMs to produce a full coverage classifier with overall improved accuracy. The hybrid approach is based on the simple idea of "punting" from one method to another using a learned threshold.

**Conclusion:**

In cross-validated experiments on the SCOP hierarchy, the hybrid methods consistently outperform the individual component methods at all levels of coverage.

Code and data sets are available at

## Background

To facilitate the automatic annotation of newly sequenced proteins or newly resolved protein structures, we are interested in developing computational methods to automatically assign proteins to structural and functional categories. Traditional computational methods for comparing protein structures depend on pairwise structural alignment programs such as CE [1], DALI [2] or MAMMOTH [3]. Similarly, sequence-based algorithms such as Smith-Waterman [4], BLAST [5], SAM-T98 [6] and PSI-BLAST [7] assign similarity scores to pairs of protein sequences. Using pairwise structural comparisons of a query sequence or structure against a curated database, one can use any of these tools to implement a nearest neighbor (NN) strategy to classify the query.

In 1999, Jaakkola *et al. *[8] first applied the support vector machine (SVM) classifier [9] to the problem of predicting a protein's structural class from its amino acid sequence. They focused on a particular protein structural hierarchy called the Structural Classification of Proteins (SCOP) [10], and they trained SVMs to recognize novel families within a given superfamily. This seminal work led to the development of many SVM-based protein classifiers (reviewed in [11]), and this work continues up to the present [12-15].

Primarily, these classifiers differ in their *kernel functions*. In this context, a kernel is a function that defines similarities between pairs of proteins. For this task, a good kernel function is one that allows the SVM to separate proteins easily according to their SCOP categories. In the experiments reported here, we train SVMs to classify amino acid sequences into SCOP superfamilies using the profile kernel [16], which is among the best-performing SVM-based methods.

More recently, several groups have extended SVM-based methods to the classification of protein structures, rather than protein sequences [17-19]. In the current work, for prediction of SCOP superfamilies from structures, we train SVMs using a kernel function based on MAMMOTH [3]. Benchmark experiments have shown that SVM-based discrimination with a MAMMOTH kernel outperforms several other SVM-based methods and also outperforms using MAMMOTH in a nearest neighbor fashion [19].

In this work, we aim to address a fundamental limitation of any SVM-based method, namely, that an SVM can only be trained when a sufficient number of training examples are available. In particular, to train an SVM to recognize a given SCOP category, we must be able to present to the SVM at least a handful of representative proteins. For under-represented SCOP categories, the SVM cannot be trained, and as a result, the classifier has limited coverage. For example, in SCOP version 1.69, 60.2% of the superfamilies contain three or fewer proteins. Failing to make predictions for these small superfamilies significantly decreases the effective accuracy of the SVM-based method, making it impractical for automated classification of the entire SCOP hierarchy.

In this study, we develop a hybrid machine learning approach that we apply to the problems of classifying proteins from sequence or from structure. Our goal is to combine nearest neighbor methods, which in principle have complete coverage over any given data set, with higher accuracy but reduced coverage multiclass SVM approaches to produce a full coverage method with overall improved accuracy. The hybrid approach is based on the simple idea of "punting" from one method to another. We use held-out data to learn a set of score thresholds. At test time, predictions from the primary method that receive scores below the threshold are "punted" to the secondary method. In addition, we consider different coverage thresholds at which to punt out of the secondary method (i.e., abstain from making a prediction altogether), and we compute error rates of the hybrid method at these different coverage levels.

We use this punting method to build hybrid predictors of SCOP superfamilies, taking as input either protein sequences or structures. Using punting, we find that the hybrid methods consistently outperform the individual component methods at all levels of coverage.

## Results

### Approach

**Figure 1:**
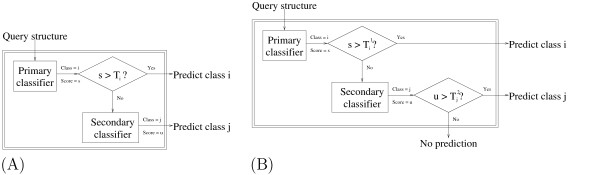
**Two punting strategies**. (A) Two classifiers are combined to produce a hybrid classifier with improved accuracy and coverage. The punting thresholds (**T **= [*T*_1_, ..., *T*_*n*_]) are class-dependent and are set using held-out data. (B) This approach is similar to (A), except that using two vectors of punting thresholds – **T**^1 ^for the primary classifier and **T**^2 ^for the secondary classifier – allows the method sometimes to make no prediction at all.

The punting strategy is depicted in Figure 1. In its simplest form (Figure 1A), the strategy relies upon a vector **T **of class-specific parameters. These parameters are learned by the algorithm, given a *single *hyperparameter supplied by the user. A query protein representation is first given to the primary classification method. The classifier produces a predicted classification *i *along with a score *s*, the magnitude of which indicates the confidence in the prediction. If this score exceeds *T*_*i*_, then the current class is predicted. Otherwise, the query is punted to the secondary classifier, which makes its own prediction. Typically, the primary classifier is the one with higher accuracy and lower coverage, although we also experiment with punting in the other direction. It is sometimes preferable to make no prediction at all, rather than make a prediction that is very likely incorrect. In this case, a second set of class-specific thresholds allows the second classifier to punt as well, as shown in Figure 1B.

**Figure 2:**
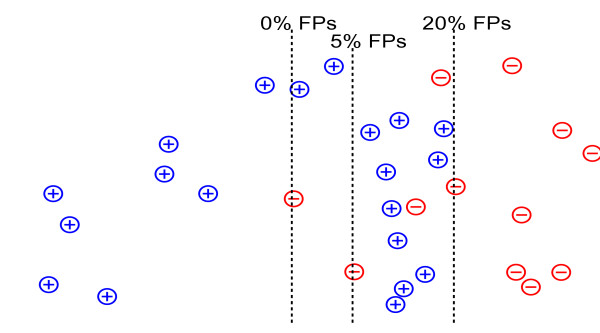
**Learning punting thresholds**. The punting threshold is learnt according to the percentage of false positives in a validation set. The figure illustrates, for a simulated data set of 20 positive and 20 negative examples, three choices of threshold: 0%, 5% or 20% false positives.

To learn punting thresholds, we divide our training set into two portions, a *classifier training set *and a *threshold training set*, which are used, respectively, to train the classifier and to learn class-specific score thresholds. The user must set a *single *hyperparameter *ρ *between 0 and 1, which controls the fraction of examples that one wishes to cover, as illustrated in Figure 2. The algorithm then sets, for each class, the score threshold such that a fraction *ρ *of the negative examples from the threshold training set are false positives, given the predictions of that classifier. Hence, when we set *ρ *= 1 the method will never punt. When we set *ρ *= 0, on the other hand, the algorithm is rather unlikely to produce a false positive. Values of *ρ *between 0 and 1 yield behavior between these two extremes.

We compare this method to a few simple variants. First, we can apply punting to a *single *method, rather than a hybrid method. In this setting, when the punting algorithm decides to punt, there is simply no prediction made at all. Second, for a given method, rather than having a vector of class-specific score thresholds **T**, we can use a single threshold that applies to all of the classes predicted. This threshold is selected so that the class-specific SVMs collectively achieve the user-specified coverage on the threshold training set. The motivation for this simpler thresholding strategy is to reduce the risk of overfitting on the threshold training set. If the confidence scores are well calibrated, then this single threshold approach should also perform well; conversely, if the scores are not well calibrated then the multi-threshold method should perform better.

### Experimental design

We tested two methods for predicting SCOP superfamilies. In the first, we made predictions from amino acid sequences, and in the second we made predictions from protein structures. For prediction from amino acid sequence, we used pairwise alignments based on PSI-BLAST for the nearest neighbor method, and we used the profile kernel [16] to define a kernel representation. For prediction from protein structure, we used structural alignments based on MAMMOTH, both for the nearest neighbor method and to define a kernel representation for training SVMs to recognize SCOP superfamilies [19]. For simplicity, in both cases we used a standard one-vs-all approach for making multiclass predictions from binary SVM classifiers.

We divided the data set (all of SCOP version 1.69) into four parts: *A*_*trn*_, *A*_*tst*_, *B*_*trn*_, *B*_*tst*_. We determined *A*_*trn *_and *A*_*tst *_to suit the requirement of training and testing binary SVM superfamily classifiers: *A*_*tst *_consists of totally held-out families from superfamilies that have 2 or more member families of at least 3 proteins each; *A*_*trn *_consists of all other families belonging to these superfamilies. Data set *B *consists of all superfamilies in SCOP that are not covered by data set *A*. *B *is then split into train and test by families at random such that the ratio of families for *B*_*tst*_/*B*_*train *_is equal to the ratio *A*_*tst*_/*A*_*train*_. The data set for superfamily detection has 74 superfamilies in *A *and 1458 superfamilies in *B *(total 1532).

We considered punting both from SVMs to the nearest neighbor method and vice versa. When using SVMs as the primary method, we used *B*_*trn *_as additional negative examples on which to calculate punting thresholds. In the reverse case, because the nearest-neighbor method had accrued no bias in "training," we used all of the negative superfamilies in *A*_*trn *_and *B*_*trn *_to determine thresholds.

### Punting once

Initially, we evaluated superfamily detection performance at full coverage, that is, when we make a prediction for every test example (as in Figure 1A). Results for classification from sequence are shown in the left half of Table 1. Here *A*_*tst *_consists of held-out families from superfamilies within the coverage of the SVM classifiers; *B*_*tst *_consists of familes outside of SVM coverage. Consequently, the SVM yields a 100% error rate on *B*_*tst*_, whereas PSI-BLAST incorrectly classifies 55.7% of these sequences. Conversely, for the sequences within classes covered by the SVM, PSI-BLAST's error rate (49.1%) is significantly higher than the SVM's error rate (24.0%). When we combine the two methods, the overall error rate drops by 10.8% from 51.8% for PSI-BLAST to 40.8% for PSI-BLAST → SVM. To evaluate the statistical significance of the observed differences in performance, we use McNemar's test to compute a *p *value for the null hypothesis that the same proportion of proteins are correctly classified by both methods. These tests, applied to the entire test set, show that each of the hybrid classifiers performs significantly better than each of the single classifiers; i.e., all four relevant *p *values are less than 0.01.

**Table 1:** Superfamily detection error rates at full coverage.

Classifying sequences					Classifying structures				
Primary	Secondary	*A*_*tst*_	*B*_*tst*_	*A*_*tst *_+ *B*_*tst*_	Primary	Secondary	*A*_*tst*_	*B*_*tst*_	*A*_*tst *_+ *B*_*tst*_
SVM	-	0.2396	1.0000	0.5510	SVM	-	0.2194	1.0000	0.5391
PSI-BLAST	-	0.4914	0.5569	0.5182	MAMMOTH	-	0.2922	0.3309	0.3081
SVM	PSI-BLAST	0.2376	0.5598	0.4322	SVM	MAMMOTH	0.1790	0.3367	0.2794
PSI-BLAST	SVM	0.2730	0.5569	0.4078	MAMMOTH	SVM	0.2053	0.3309	0.2633

Each entry in the table is the fraction of proteins in the test set that are assigned to the incorrect superfamily by the given method.

Results from the classification of protein structures are shown in the right half of Table 1. For this task, a drop in error rate of 4.5% (30.8% to 26.3%) is achieved from MAMMOTH to MAMMOTH → SVM. Again, McNemar's test shows that both hybrid methods outperform both of the single classifiers at *p <*0.01.

### Punting once versus punting twice

In practical applications, it may be preferable for the classifier to say "I don't know" rather than return an incorrect classification. To achieve this behavior, we included a second level of punting, based on a second set of thresholds (Figure 1B). This strategy allows the classifier to punt completely and not give a prediction for an example. The target percentage for both the primary and final punting thresholds were varied for both hybrid methods, yielding a range of coverage and error rates.

**Figure 3:**
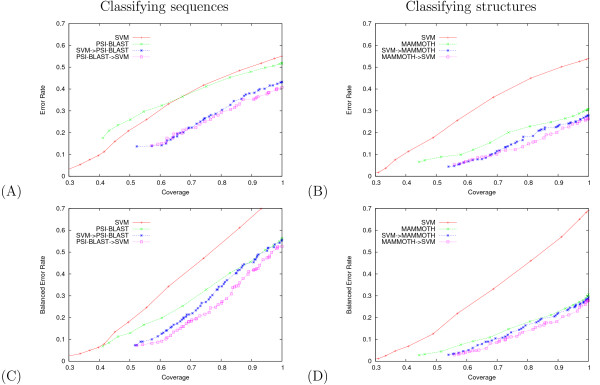
**Hybrid methods for protein classification yield lower error for all coverage values**. The figures plot (A, B) unbalanced error rates and (C, D) balanced error rates as a function of unbalanced coverage. In panels (A) and (C), we are classifying protein sequences; in panels (B) and (D), we are classifying protein structures.

Results for classification over a range of coverages can be seen in Figure 3A for protein sequences and Figure 3B for protein structures. For both tasks, punting in either direction – from the SVM to the nearest neighbor classifier or vice versa – yields higher accuracy than either single method at all coverage rates. The unbalanced error rate used in Figure 3A–B counts the number of proteins in the test set whose SCOP superfamily is incorrectly predicted; hence, this metric implicitly assigns more weight to larger classes. To evaluate the improvement over small classes, we also measured the balanced error rate, in which we compute the error rate separately for each class and then average the resulting values (Figure 3B–C). Again, punting in either direction, we generally achieve higher balanced accuracy with the hybrid method for both classification tasks.

**Figure 4:**
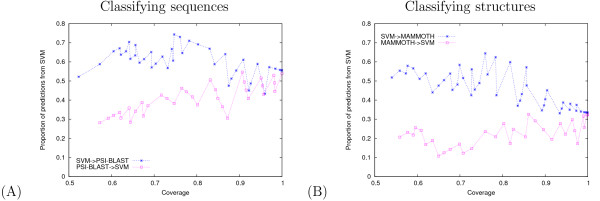
**Percentage of predictions made by the SVM**. Each panel plots, for one of the two classification tasks, the percentage of predictions made by the SVM as a function of the total number of predictions made.

To understand better why the punting procedure produces better overall accuracy, we plot in Figure 4 the percentage of predictions made by the SVM as a function of the total number of predictions. The oscillatory behavior of all four series is a result of the grid search over two independent punting thresholds. The hybrid classifier can either (1) assign a low threshold to punt from method A to method B and a high threshold to make no prediction or (2) assign a high threshold to punt from A to B and a low threshold to make no prediction. These two strategies achieve a similar level of coverage and a similar errror rate but, as shown in Figure 4, the resulting set of predictions may contain quite different percentages of predictions from each of the individual classifiers.

Comparing Figure 4A and 4B, we see a different overall trend for the two classification tasks. For the sequence classification problem, as coverage approaches 100%, the two methods end up sharing predictions almost 50/50. In contrast, for the structure classification problem, the SVM method converges to fewer predictions – MAMMOTH makes approximately twice as many predictions as the SVM at full coverage. This observation may explain why the improvement provided by the hybrid classifier is smaller in the structure classification problem (4.5% decrease in error) compared with the sequence classification problem (10.8% decrease). For the structure classification task, the high coverage classifier (MAMMOTH) is already very good, so adding a second, supervised classifier does not yield a large improvement.

### Single versus multiple thresholds

**Figure 5:**
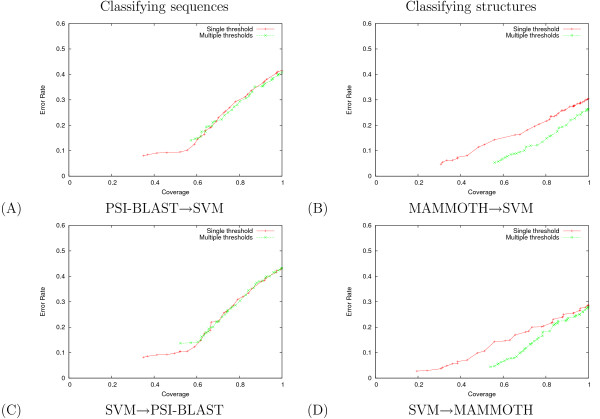
**The value of using class-specific thresholds**. The figure compares, for both hybrid methods on both classification tasks, the performance when using a single threshold for all classes versus using class-specific *learnt *thresholds.

Thus far, we have reported results using class-specific thresholds. A simpler approach would be to learn a single, class-independent threshold for a given classifier. Figure 5 compares the results of these two approaches for the hybrid methods on both classification tasks. For classification of protein structures, using class-specific thresholds consistently improves the overall performance. In contrast, when we apply the same analysis to classification by sequence, we find there is no benefit from using multiple thresholds. Using multiple thresholds should help when the class-specific classifiers are not well calibrated; i.e., when an observed score of *X *always corresponds to the same class-conditional posterior probability. Thus, these results suggest that the E-values returned by MAMMOTH are not as well calibrated as those computed by PSI-BLAST.

### Combining low and high coverage methods

As mentioned above, approximately 60% of the SCOP superfamilies in our data set contain fewer than three members. The punting methodology allows us to predict members of these superfamilies, even though an SVM is not trained for small superfamilies. Moreover, if the high coverage (NN) classifier incorrectly places a member of a large superfamily into a small superfamily, then the low coverage classifier (SVM) can correct this error, because it has high accuracy for large superfamilies.

**Figure 6:**
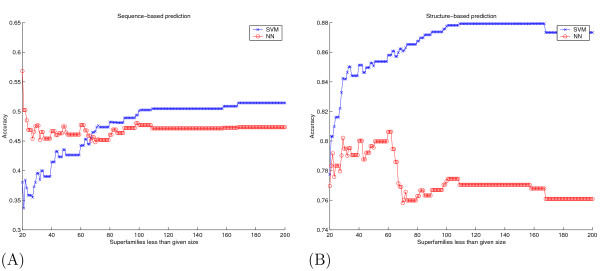
**SVM performs better than NN for larger superfamily sizes**. The figure plots the accuracy of NN and SVM classifiers (y-axis), averaged over all superfamilies less than or equal to a given size (x-axis) for classification from (A) sequence and (B) structure.

An alternative to the approach described here would be to attempt to train an SVM even for superfamilies with one or two members. In this case, we could still punt from the SVM to NN or vice versa. We do not expect, however, this approach to yield a significant improvement, because SVMs are not designed to work well from so few examples. Figure 6 provides evidence to support this claim. For both sequence and structure based prediction experiments, we plot the accuracy for SVMs over NNs averaged over all superfamilies less than or equal to a given size. One can see that as the superfamily size increases, the accuracy gain of SVM over NN increases. For the sequence-based prediction problem, for small superfamily sizes, SVM is on average outperformed by NN. For example, the average accuracy of SVM and NN for all superfamilies less than size 30 is 0.3956 and 0.4762 respectively. In contrast, for all superfamilies *larger than *size 30 the averages are 0.6578 and 0.4714 respectively.

If the effect shown in Figure 6 were not a problem – i.e., if both classifiers worked well enough across all superfamily sizes – then one could use standard methods for combining classifiers, such as a voting scheme. However, even in such a case, one would still not be able to control the accuracy versus coverage of predictions. This flexibility, which is provided by the punting strategy, is one of the main contributions of our work.

### Punting as stacked generalization

Stacked generalization [20] is a general scheme for optimizing the generalization error of several classifiers by learning how to combine them accurately. The basic idea is to (i) train each classifier on the same problem and then (ii) use a second set of data to learn a combining scheme when using these classifiers. One example of this approach is that in stage (ii) one could construct a feature space whose inputs are the guesses of the classifiers trained in stage (i), so training a linear classifier in stage (ii) would mean learning a weighted majority vote over the classifiers. However, the stacked generalization approach, as Wolpert describes it, can include any two-stage method of combination. In that sense, our punting method is an instance of stacked generalization where the second stage learns a function that chooses which classifier to apply, depending on the magnitude of the real-valued outputs (i.e., the classifier decides when to punt). Just as in stacked generalization, we divide our data set into two portions: one for training stage (i), the classifiers, and one for training stage (ii), the punting thresholds. However, Wolpert neither describes the use of punting for choosing classifiers, nor for finding a trade-off between coverage and accuracy of the resulting combined classifier, making our approach a novel instance of his general scheme.

### AutoSCOP comparison

We compared the performance of our hybrid classifiers with that of the webserver AutoSCOP [21].

AutoSCOP uses a database built from SCOP 1.69, as does our method. To test both methods, we therefore created a dataset of 100 new protein domains from SCOP version 1.73. We combined the dataset used in this study, consisting of 11,944 sequences from Astral version 1.69, with 9,536 sequences from Astral version 1.73, and we clustered the combined set using a 40% sequence identity threshold. We then identified clusters that contained only sequences from version 1.73, and we extracted the longest sequence from each of these clusters. This procedure yielded a total of 2285 novel domain sequences, which are members of 698 distinct SCOP superfamilies. Finally, we randomly selected 100 of these sequences for use in our test. Results for superfamily detection can be found in Table 2. Our simple hybrid classifier achieves a 27% error rate, which is nearly as good as the 25% error rate achieved by AutoSCOP. Most of the difference between the methods arises for small superfamilies, where the SVM is not applicable. For superfamilies that are covered by an SVM, the SVM error rate (3/38 = 7.9%) is less than half AutoSCOP's error rate (7/38 = 18.4%).

**Table 2:** AutoSCOP comparison.

Method	Covered	Uncovered	Total
AutoSCOP	7	18	25
SVM	3	62	65
PSI-BLAST	6	24	30
SVM → PSI-BLAST	4	40	44
PSI-BLAST → SVM	3	24	27
Total	38	62	100

Each entry in the table is the number of proteins from a test set of 100 proteins taken from SCOP.1.73 that are assigned to the incorrect superfamily by the given method. Each superfamily is "covered" or "uncovered," depending on whether an SVM has been trained to recognize it.

## Discussion

We have described a simple method of combining a high coverage, low accuracy classifier with a low coverage, high accuracy classifier, based on learning a collection of class-specific thresholds from held-out data. For SCOP superfamily recognition from structure and sequence, the resulting hybrid classifiers yield consistently lower error rates across a wide range of coverage.

*A priori*, punting seems most intuitive when the low-coverage/high-accuracy classifier punts to the high-coverage/low-accuracy classifier. However, the results in Figure 3 suggest that, for the combination of SVM and NN classifiers applied to SCOP classification, punting in the opposite direction is slightly more effective. We speculate that the best performance will be obtained when the primary classifier is the one that returns the most accurate *confidence measure *in its predictions, rather than the most accurate generalization performance. In this way, if the primary classifier always punts accurately when it is incorrect, then the combined generalization performance can be optimized. Hence, the NN → SVM hybrid may be slightly better than the SVM → NN hybrid because the NN method punts more accurately.

One of the primary contributions of this work is to make SVM-based classifiers practically applicable. Although they have been shown to provide superior performance for protein classification problems in which the number of examples is large enough, SVMs have not been used in practice because of their limited coverage. On the other hand, the goal of this paper is not to argue that SVMs are better than other methods, but to show how to make an SVM classifier practical, by giving it complete coverage. Our results presumably generalize to other supervised classification algorithms, though we have not tested this hypothesis directly.

For simplicity of exposition, we have used a simple one-vs-all approach to multiclass SVM classification. In practice, it is generally preferable to use a more complex multiclass approach such as code learning [13]. Combining code-learning with the punting approach described here yields even lower error rates than are shown in Figure 3 (data not shown). In general it is straightforward to combine any pair of (low and high coverage) classifiers using our approach. The only prerequisite is that they provide a real-valued output for each class, and that these values are correlated with the confidence in their predictions. From these outputs we can learn punting thresholds.

In this work, we use a relatively simple strategy to define the data for learning punting thresholds given the user-specified hyperparameter *ρ*. More complex internal cross-validation schemes would likely yield slightly better performance and increased running time.

Eventually, rather than combining two existing classifiers, we would like to train a single classifier that has the advantages of both systems in one. This approach would obviate the need for the punting strategy described here. We are currently investigating approaches to this problem by training a ranking based algorithm, rather than a class predictor.

## Authors' contributions

The authors jointly conceived and designed the experiments and wrote the manuscript. Iain Melvin carried out the experiments.
